# The role of mother-youth biobehavioral synchrony during stress in predicting youth well-being

**DOI:** 10.3389/frcha.2026.1923350

**Published:** 2026-10-02

**Authors:** Christina A. Rowley, Juelle Ford, Frances Haofei Li, Zahara O. Cuevas-Kovanis, Monet Ruiz, Jennifer A. Somers, Jessica L. Borelli

**Affiliations:** 1 Psychology Department, The Health, Relationships, and InterVEntions Lab (THRIVE), University of California, Irvine, CA, United States; 2 Psychological Sciences Department, Families: Leveraging Opportunities for Wellbeing and Resilience (FLOWER) Laboratory, Auburn University, Auburn, AL, United States

**Keywords:** cardiovascular, mental health, parent-child, physiological, stress, synchrony

## Abstract

**Introduction:**

Parent-child physiological synchrony may reflect coordinated regulatory processes, but its meaning appears to vary across developmental and interactional contexts. Greater synchrony has been associated with more and less adaptive functioning, suggesting that synchrony should not be interpreted as inherently beneficial. The current study examined whether mother-youth trend-level physiological synchrony during a dyadic stressor was associated with multiple indicators of wellbeing in preadolescence.

**Method:**

One hundred seventy-three mother-child dyads (Mothers: *M_age_* = 41.60 years; Youth: *M_age_* = 9.54 years, range = 8–12; 53% female) completed the Performance Challenge Task, during which youth attempted unsolvable puzzles while mothers observed. Respiration rate, root mean square of successive differences (RMSSD), and respiratory sinus arrhythmia (RSA) were assessed simultaneously in mothers and youth. Youth reported positive and negative affect, depressive symptoms, anxiety, and aggression; mothers reported youth internalizing and externalizing symptoms. Growth models estimated each participant's physiological trajectory across the task, and mother and youth trajectories were covaried to estimate trend-level synchrony.

**Results:**

Greater RSA synchrony was associated with higher post-stressor positive affect after accounting for pre-stressor positive affect, higher mother-reported internalizing symptoms, and lower mother-reported externalizing symptoms. RMSSD synchrony showed a nonsignificant trend toward higher youth-reported anxiety (*p* = 0.08), and respiration-rate synchrony showed a nonsignificant trend toward lower mother-reported externalizing symptoms (*p* = 0.06). Most other associations were nonsignificant.

**Discussion:**

Findings did not support a uniformly adaptive interpretation of physiological synchrony. Instead, associations varied across outcomes and physiological indices, underscoring the importance of considering what form of synchrony is measured and the context in which it occurs.

## Introduction

Feldman's (1) biobehavioral synchrony theory proposes that coordination in parents' and children's physiology, behavior, and affect contributes to affiliative bonds and the development of regulatory capacities (1–5). Co-regulation, more broadly, refers to the bidirectional ways in which interaction partners influence one another's regulatory states over time (6). Physiological synchrony, covariation in dyad members' physiological responses, may serve as a marker of these dyadic processes. Importantly, however, physiological synchrony is an observed pattern of coordination rather than direct evidence of co-regulation; depending on the context, it may reflect adaptive coordination, shared arousal, mutual dysregulation, or other dyadic processes.

### Parent-child synchrony in context

The meaning and correlates of physiological synchrony appear to depend on characteristics of the dyad and the interactional context, including parent and child psychopathology, trauma exposure, and the regulatory demands of the situation (5, 7). Although greater parent-child physiological synchrony has been associated with better socioemotional adjustment in some studies (4), synchrony observed under conditions of distress has also been linked to poorer regulatory functioning (2). During stress, positive synchrony may reflect shared physiological arousal or stress contagion rather than effective regulation (8–10). Conversely, divergent physiological responses may sometimes be adaptive: a caregiver who remains regulated while a child becomes increasingly aroused may be better positioned to support the child's regulation (4, 7, 8). Thus, physiological synchrony is not inherently adaptive or maladaptive. Its interpretation calls for attention to the demands of the interaction and to the specific physiological process being assessed. The current study extends this work by examining multiple indices of mother-youth synchrony in response to a standardized stressor.

### Indices of physiological stress

Studies of parent-child physiological synchrony commonly rely on a single physiological index [e.g., respiration rate (7, 11)], which may provide an incomplete account of dyadic physiological coordination. Different autonomic indices reflect partly distinct physiological processes and may therefore show different patterns of association with child functioning. The autonomic nervous system (ANS) comprises the sympathetic (SNS) and parasympathetic (PNS) branches that together mediate responses to environmental demands (12). SNS activation generally supports physiological mobilization during challenge or threat, whereas the PNS contributes to regulation and recovery following stress (12, 13). Because autonomic measures differ in the extent to which they reflect sympathetic and parasympathetic influences, examining multiple indices may help clarify whether findings generalize across physiological functions.

Respiration rate reflects broad autonomic activation and has been used in studies of parent-child physiological synchrony (7, 11). Respiratory sinus arrhythmia [RSA (14)] and the root mean square of successive differences [RMSSD (15)], by contrast, are heart rate variability measures commonly used as indices of parasympathetic cardiac activity. Higher RSA and RMSSD generally reflect greater parasympathetic influence, although the measures are not interchangeable and are affected differently by respiration and other methodological factors (16–20). RSA is closely tied to respiration, whereas RMSSD is comparatively less affected by respiratory rate (17–20). Examining respiration rate, RSA, and RMSSD in the same dyads therefore permits comparison of synchrony across a broader autonomic index and two closely related indices of PNS-mediated functioning.

Physiological synchrony can also be evaluated at different timescales (21). Trend synchrony captures similarity in dyad members' overall patterns of physiological change over the course of an interaction. Concurrent synchrony captures whether momentary deviations in one person's physiology coincide with deviations in the other person's physiology. In contrast, lagged synchrony assesses whether changes in one partner precede changes in the other. These approaches address different questions related to physiological synchrony. Still, concurrent and lagged synchrony are more similar to each other than trend synchrony since they both measure coordination at the moment-to-moment level, whereas trend-level synchrony captures coordination across time. The current study focuses on trend-level synchrony, as it characterizes systematic changes in physiological responses over time, allowing us to understand the extent to which each parent and their child exhibit similar patterns of physiological change during a stressor.

### Parent-child relational dynamics in developmental context

Parent-child co-regulation changes substantially across development. Early in life, children depend heavily on caregivers for physiological and emotional regulation (22, 23). With development, children acquire increasingly independent regulatory capacities, although caregivers remain important sources of support. Difficulties with self-regulation during childhood and adolescence are associated with a range of internalizing and externalizing problems (24–27), pointing to the importance of understanding how dyadic physiological coordination relates to functioning beyond early childhood. Existing research has linked parent-child physiological synchrony with outcomes including internalizing symptoms, parenting behavior, and empathy (28–31), but findings are not uniformly consistent and vary across contexts.

Middle childhood and preadolescence are especially relevant periods for examining these processes because, during these phases, youth develop greater autonomy while continuing to rely on caregivers under stress (32). The parent-child relationship increasingly takes the form of a supervisory partnership in which psychological availability, communication, and collaborative decision-making become more prominent than physical proximity (33). Parents nevertheless remain important attachment figures during this period, particularly when youth are distressed or seek help (34).

These developmental changes may alter the form and meaning of physiological coordination between parents and youth. Compared with younger children, preadolescents may regulate more independently even while drawing on parental support, potentially lessening the extent to which physiological similarity reflects caregiver-led regulation. Yet most research on parent-child physiological synchrony has focused on infancy and early childhood (5, 7, 35). Examining synchrony during preadolescence can therefore clarify whether associations observed earlier in development extend to a period characterized by greater regulatory autonomy.

The current study examined whether mother-youth trend-level physiological synchrony during a standardized cognitive stressor was associated with youth emotional and behavioral functioning. Specifically, we examined synchrony in respiration rate, RSA, and RMSSD in relation to (1) post-stressor positive and negative affect, accounting for pre-stressor affect; (2) youth-reported anxiety, depressive symptoms, and aggression; and (3) mother-reported internalizing and externalizing symptoms. Prior findings provide reasons to expect both adaptive and maladaptive correlates of synchrony during stress. Based on studies linking greater parent-child synchrony with more adaptive functioning, we tentatively expected greater synchrony to be associated with higher post-stressor positive affect, lower post-stressor negative affect, and fewer emotional and behavioral symptoms. However, because prior work has largely examined moment-to-moment rather than trend-level synchrony, these hypotheses were considered exploratory.

## Methods

### Participants

Dyads were eligible if the child was 8–12 years old, the mother and child were both proficient in English, the mother and child lived together, and the child had not been diagnosed with a developmental disorder. Participants were 173 mothers (*M_age_* = 41.60 years, *SD* = 5.48, Range = 29–61) and their children (53% female; *M_age_* = 9.54 years, *SD* = 1.33, Range = 8–12). Self-reported race and ethnicity were White (33%), Asian (31%), Latine (25%), Mixed (8%), Native Hawaiian/Pacific Islander (1%), Native American/Indigenous (1%), and Other (1%). Most mothers had completed a graduate degree (41%) or bachelor's degree (37%); 11% reported some college, 9% an associate's degree, and 2% a GED. Median annual household income was $120,000-$129,000. Participants were recruited through a multi-laboratory participant pool, social media, and community settings such as after-school programs. The sample size was determined by the design of the larger IRB-approved study (IRB #20173772), which included three experimental conditions.

### Study design

The current project used data from a larger study of mothers and children conducted between 2017 and 2026. After consent and assent, dyads completed questionnaires, semi-structured interviews, and laboratory tasks. Before the stressor task, mothers were randomly assigned to one of three memory-task conditions: relational savoring (36), an academic condition focused on the child's academic strengths and weaknesses, or a control condition focused on the mother's weekly schedule. Condition was not a focus of the present study and was included as a covariate in analyses. Youth then completed the Performance Challenge Task (PCT; 36), consisting of six 60-second computer-based puzzles adapted from the Block Design subtest of the WISC-III (37). All puzzles were unsolvable, although mothers and youth were not told this. Mothers observed and were instructed: “only help if you think he/she [referring to their child] really needs it.” Electrocardiogram (ECG) data were recorded continuously from both dyad members. Before the PCT, mothers and youth separately watched a nature video to provide a neutral baseline. PCT data were segmented into six 60-second epochs corresponding to the six puzzles (see Figure 1 for illustration of study procedures and timeline). Physiological data collected after the PCT were not analyzed in the present study.

**Figure 1:**
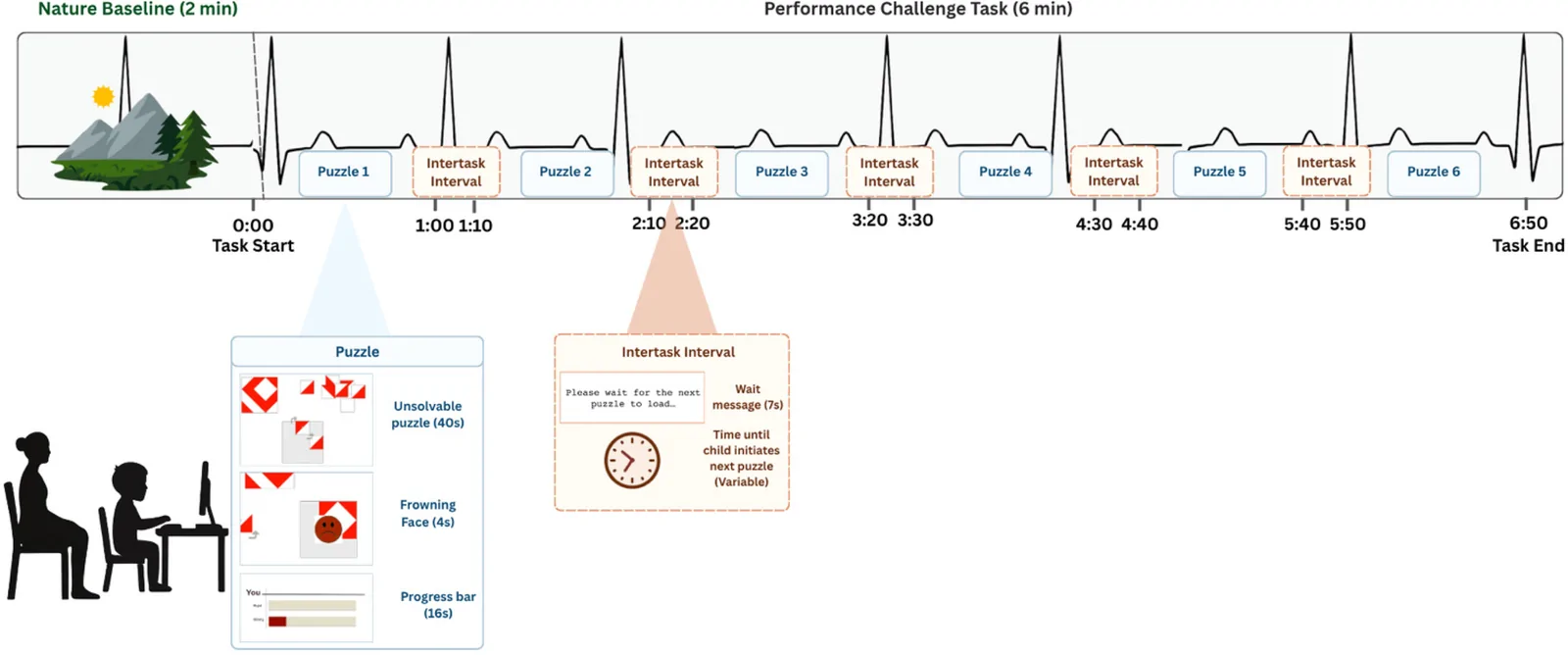
Illustration of nature baseline and performance challenge task timeline and procedures. Electrocardiogram (ECG) data were recorded continuously throughout the 2-minute nature baseline and the 6-minute performance challenge task as illustrated by the waves layered behind these two study events. ECG data were segmented into 6 consecutive 60-second epochs anchored to each puzzle onset. The roughly 10-second intertask intervals were excluded from analyses. The total analyzed task duration was 6 min.

### Measures

#### Youth well-being

##### Emotion

The Positive and Negative Affect Schedule for Children [PANAS-C (38)] was used to assess children's positive and negative state affect. The PANAS-C is an eight-item self-report measure in which youth rated the extent to which they experienced various emotions (e.g., joyful, sad, nervous, calm) in that exact moment. The PANAS-C was administered before and after the PCT and rated on a 5-point scale (1 = *very slightly or not at all*; 5 = *extremely*). Internal consistency was adequate for pre-stressor (positive affect *α* = 0.67; negative affect *α* = 0.79) and post-stressor (positive affect *α* = 0.64; negative affect *α* = 0.76).

##### Depressive symptoms

The Children's Depression Inventory [CDI (39)] assessed behavioral, cognitive, emotional, and physical symptoms of depression in the past two weeks in children. It includes 27 items scored from 0 to 2, with higher scores representing statements indicating greater depression. For each item, the youth selected 1 of 3 statements (e.g., “I am sad once in a while,” “I am sad many times,” “I am sad all the time”). Higher scores indicate greater depressive symptoms. Internal consistency was adequate (*α* = 0.88).

##### Anxiety symptoms

The Multidimensional Anxiety Scale for Children [MASC (40)] is a 29-item self-report questionnaire that was used to assess recent anxiety symptoms (physical, social anxiety, harm avoidance, and separation anxiety) in children. Children rated the frequency of anxiety-related symptoms (e.g., “I get shaky or jittery”) on a 4-point scale (0 = *never* to 3 = *often*). Higher scores indicate greater anxiety symptoms. Internal consistency was adequate (*α* = 0.87).

##### Aggression

The Aggression Questionnaire [AQ (41)] consists of 34 items measuring children's aggressive behaviors and attitudes, including physical aggression, hostility, verbal aggression, indirect aggression, and anger. Each item is rated by youth on a 5-point scale (1 = *not at all like me* to 5 = *completely like me*). Higher scores indicate greater aggression. Internal consistency was adequate (*α* = 0.90).

##### Internalizing and externalizing behavior

Mothers completed the Child Behavior Checklist for children ages 6–18 years old [CBCL/6–18 (42)] rating the extent to which their child displayed different internalizing and externalizing behaviors (e.g., “impulsive or acts without thinking”) in the past 6 months on a 3-point scale (0 = *not true* to 2 = *very true or often true*). Higher scores reflect greater behavioral and emotional difficulties. Internal consistency was adequate (externalizing *α* = 0.85; internalizing *α* = 0.86).

#### Physiological measures

Physiological activity was recorded simultaneously from mothers and youth during the PCT using Mindware equipment. Seven electrodes were placed on the front and back of the participant's chest and torso in a tetrapolar 7-spot configuration, which includes a modified Lead II ECG configuration and four electrodes for impedance cardiography. Disposable pediatric 1 ⅝-inch foam electrodes with 1% chloride wet gel were used for children, and disposable adult 1.5-inch foam electrodes with 7% chloride wet gel were used for mothers. Signals were collected using Mindware Mobile and Biolab Software 3.3.1. These procedures were all done prior to the start of the PCT. Respiration rate was examined as an index of broader autonomic functioning. RMSSD and RSA were examined as indices of PNS-mediated functioning. The HF/RSA band was set to standard adult (0.12–0.40 Hz) and child (0.24–1.04 Hz) frequency ranges, thereby allowing HF/RSA to serve as an index of respiration rate.

ECG data were processed using Mindware HRV Analysis Software 3.2.5. A trained research assistant cleaned each record, and a second research assistant reviewed the data for accuracy. Research assistants verified heartbeat detection and manually corrected artifacts, including missed beats and peak-detection errors, when necessary.

#### Analytic plan

Before hypothesis testing, we examined descriptive statistics, bivariate correlations, and demographic differences in study variables (Tables 1–5). ANOVAs indicated differences in several physiological and outcome variables by race, maternal employment, and marital status (Table 3). Bivariate analyses also identified associations involving child age, child sex, mother age, income, education, and other demographic variables. For outcome variables, there were differences by race in terms of post-stressor positive affect, internalizing symptoms, and externalizing symptoms; as well as by mothers' employment status in terms of aggression and by mothers' marital status in terms of externalizing symptoms (Table 5). In examining bivariate correlations and ANOVAs, child age, child sex, mother age, race, income, marital status, employment status, and education were significantly associated with study variables. To preserve model parsimony, child sex, mother age, and race were retained as covariates because they remained significant in the final models.

**Table 1:** Descriptive statistics for outcome variables.

Variable	N	Range	M	SD
Child-Reported				
Pre-Stressor Positive Affect	170	4–20	12.44	3.69
Post-Stressor Positive Affect	170	4–20	10.72	3.87
Pre-Stressor Negative Affect	169	4–18	5.92	2.63
Post-Stressor Negative Affect	170	3–19	6.12	2.90
Depression Symptoms	159	0–43	7.01	6.41
Anxiety Symptoms	156	13–95	50.40	13.72
Aggression	107	27–136	64.79	19.74
Parent-Reported				
Internalizing Symptoms	167	0–4	0.83	0.95
Externalizing Symptoms	167	0–29	5.59	5.28

Low scores on all variables means low levels of the measured construct; high scores on all variables means high levels of the measured construct.

**Table 2:** Relationships between covariates and cardiovascular reactivity.

Variable	Child			Mother		
	Respiration Rate	RMSSD	RSA	Respiration Rate	RMSSD	RSA
Child						
Age	0.01	0.07*	0.01	0.01	−0.02	−0.04
Sex	−0.02	0.13***	0.09**	−0.02	0.01	−0.01
Mother						
Age	0.002	0.02	0.02	0.0001	−0.21***	−0.24***
Education	0.01	0.01	0.003	−0.02	−0.12***	−0.08**
Income	0.05	0.09**	0.05	−0.11	−0.09**	−0.04

Education Level -higher numbers reflect higher education attained; Child Sex (0 = Female, 1 = Male).

† *p* < 0.10.

* *p* < 0.05.

** *p* < 0.01.

*** *p* < 0.001.

**Table 3:** Demographic group differences in cardiovascular reactivity.

Variable	Child			Mother		
	Respiration Rate	RMSSD	RSA	Respiration Rate	RMSSD	RSA
Race	*F*(5,160) = 1.22	*F*(5,160) = 2.38*	*F*(5,160) = 4.11**	*F*(5,158) = 2.99*	*F*(5,159) = 6.77***	*F*(5,159) = 4.23**
Significance *p* < 0.05			Asian and White < Mixed	Native < Other	Asian, White, and Other < Mixed	Asian and White < Mixed
Marital Status	*F*(9,162) = 0.99	*F*(9,162) = 3.37***	*F*(9,162) = 5.58***	*F*(9,161) = 7.08***	*F*(9,161) = 2.18*	*F*(9,161) = 8.35***
Significance *p* < 0.05		M < Se	C, D, M, Se, Si < W	C, D, M, Si < W; D < Se		
Employment Status	*F*(5,164) = 4.23**	*F*(5,164) = 1.61	*F*(5,164) = 3.50**	*F*(5,163) = 2.53*	*F*(5,164) = 33.41***	*F*(5,164) = 21.00***
Significance *p* < 0.05	SE < R		PT, FT > R	R < U	PT > SE, FT, U; R > PT, SE, FT, U	PT > SE, FT, U; R > PT, SE, FT, U U < PT

Marital Status (C, cohabiting, D, divorced, M, married, Se, separated, Si, single, W, widowed). Employment (FT, full-time; PT, part-time; R, retired; SE, self-employed; U, unemployed). *Post-hoc* comparisons were compared using Bonferroni corrections.

† *p* < 0.10.

* *p* < 0.05.

** *p* < 0.01.

*** *p* < 0.001.

**Table 4:** Relationships between covariates and outcome variables.

Variable	Child-Reported					Parent-Reported	
	Positive Affect	Negative Affect	Depression	Anxiety	Aggression	Internalizing Symptoms	Externalizing Symptoms
Child							
Age	−0.04	0.02	−0.02	−0.20**	−0.12	0.06	−0.03
Sex	−0.01	0.01	0.01	−0.002	0.04	−0.10	0.10**
Mother							
Age	0.01	0.02	−0.003	−0.05	0.03	−0.10	−0.08*
Education	−0.03	−0.11	−0.08	0.003	−0.08	0.02	−0.01
Income	−0.08	−0.09	−0.01	−0.001	0.11	−0.14^†^	−0.07*

Education Level -higher numbers reflect higher education attained; Child Sex (0 = Female, 1 = Male). Positive and negative affect are both post-stressor.

† *p* < 0.10.

* *p* < 0.05.

** *p* < 0.01.

*** *p* < 0.001.

**Table 5:** Demographic group differences in outcome variables.

Variable	Child					Mother	
	Positive Affect	Negative Affect	Depression	Anxiety	Aggression	Internalizing Symptoms	Externalizing Symptoms
Race	*F*(5,160) = 13.84***	*F*(5,160) = 21.56***	*F*(5,150) = 5.21***	*F*(5,148) = 6.29***	*F*(5,100) = 10.21***	*F*(5,156) = 20.64***	*F*(5,156) = 2.91*
Significance *p* < 0.05	Native > Asian, White, and Mixed; Asian and White < Other; Pacific Islander > Asian, White, Mixed, and Other	Asian and White > Mixed; Asian, Pacific Islander, White, and Mixed < Other				Native > Asian, Pacific Islander, White, Mixed, Other; Mixed > Asian, Pacific Islander, White; Pacific Islander < White, Other	Pacific Islander < Other
Marital Status	*F*(9,161) = 9.52***	*F*(9,161) = 7.25***	*F*(9,153) = 4.52***	*F*(9,151) = 6.39***	*F*(8,102) = 10.04***	*F*(9,158) = 3.37***	*F*(9,158) = 2.13*
Significance *p* < 0.05	M < Se, D	M, C, D, Si < Se	M, W < D	M, C, D, Si > Se			M < Se
Employment Status	*F*(5,162) = 1.94	*F*(5,162) = 1.71	*F*(5,153) = 6.57***	*F*(5,151) = 6.27***	*F*(5,102) = 21.47***	*F*(5,159) = 4.84***	*F*(5,159) = 4.75***
Significance *p* < 0.05			PT < FT	PT, SE, R, FT < U	U > PT, SE, FT, R		PT > R, FT

Marital Status (C, cohabiting, D, divorced, M, married, Se, separated, Si, single, W, widowed). Employment (FT, full-time; PT, part-time; R, retired; SE, self-employed; U, unemployed). *Post-hoc* comparisons were compared using Bonferroni corrections.

† *p* < 0.10.

* *p* < 0.05.

** *p* < 0.01.

*** *p* < 0.001.

To evaluate whether mother-youth trend-level physiological synchrony is associated with wellbeing during preadolescence, multilevel latent growth modeling was conducted in Mplus 8.1 (43). First, to estimate mother-youth trend-level physiological synchrony during the PCT, growth models were used to estimate within-person change in physiological stress (i.e., cardiovascular reactivity) across the six PCT epochs for mothers and youth separately. Mother and youth growth curves were then covaried to estimate the degree of similarity between their trajectories (21). Linear and quadratic growth functions were evaluated; quadratic models did not provide a better representation of change based on the slope estimates,^1^ so linear trajectories were used in the present analyses.

Then, at the between-person level, mother-youth physiological synchrony (a latent factor created using mother's and youth's physiological stress growth curves) was used as the between-person predictor of youth wellbeing (Aim 1: positive and negative affect, Aim 2: child-reported, Aim 3: mother-reported internalizing and externalizing symptoms, affect, aggression, anxiety, and depression). See Figure 2 for an illustration of the multilevel growth curve model being used in the current study. Synchrony during the neutral baseline was initially included as a covariate but was not significant; removing it did not alter the direction or significance of the focal associations, so it was omitted from the final models.

**Figure 2:**
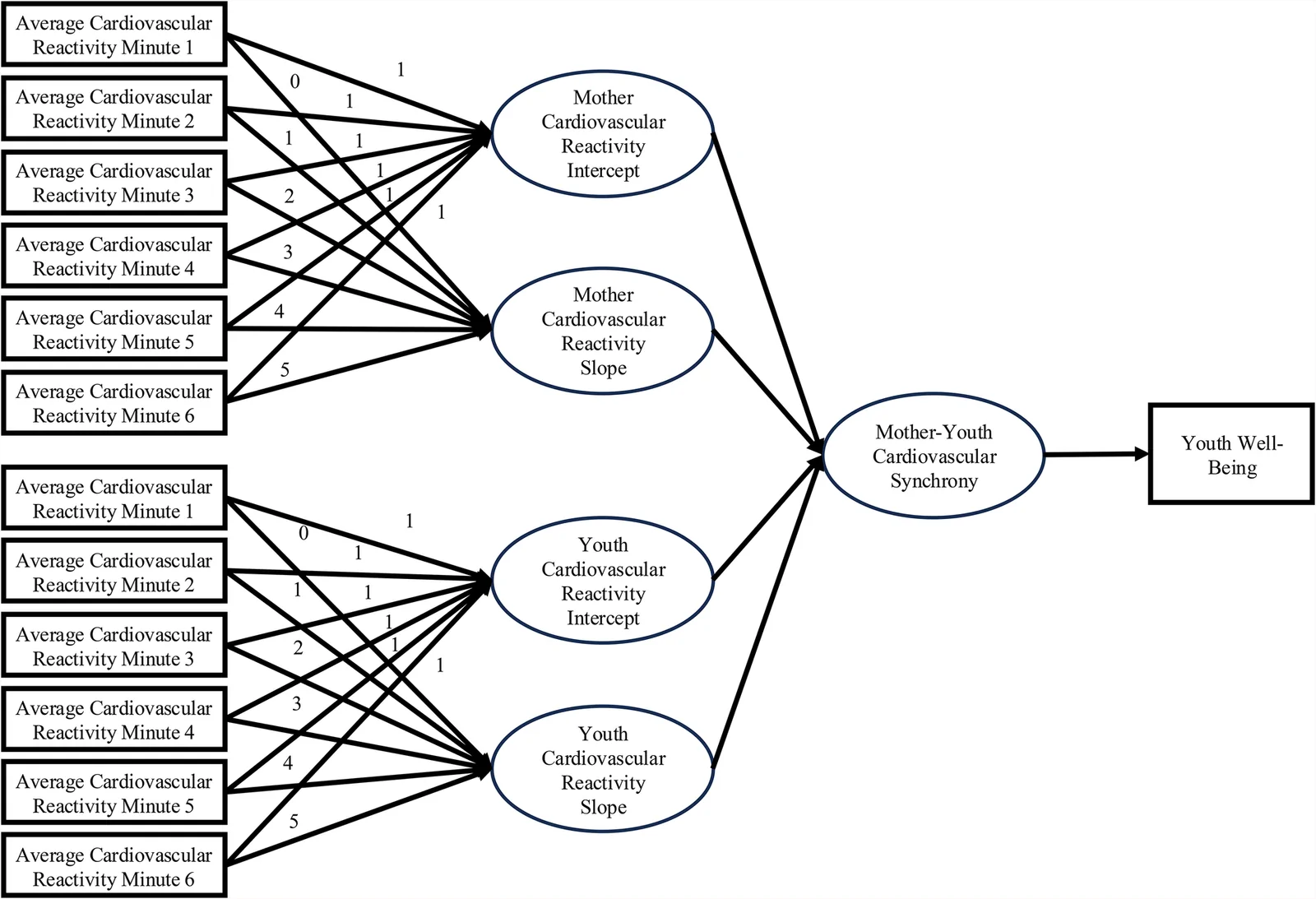
Illustration of the multilevel growth model for examining the association between mother-youth trend-level cardiovascular synchrony and youth wellbeing.

## Results

Before hypothesis testing, we examined intercorrelations among youth wellbeing outcomes and latent synchrony factors. As seen in Table 6, many youth wellbeing outcomes were modestly correlated. Pre- and post-PCT measures of the same affect dimension were more strongly associated (Table 6), indicating that these wellbeing variables represent related yet distinct measures of wellbeing. As such, we examined their associations with synchrony separately.

**Table 6:** Youth wellbeing intercorrelations.

Variable	1	2	3	4	5	6	7	8
Child-Reported								
1. Pre-Stressor Positive Affect	-	-	-	-	-	-	-	-
2. Post-Stressor Positive Affect	0.64***	-	-	-	-	-	-	-
3. Pre-Stressor Negative Affect	−0.05	−0.01	-	-	-	-	-	-
4. Post-Stressor Negative Affect	−0.03	−0.03	0.85***	-	-	-	-	-
5. Depression Symptoms	0.03	0.14***	0.14***	.04	-	-	-	-
6. Anxiety Symptoms	−0.03	−0.09**	0.13***	0.12***	0.42***	-	-	-
7. Aggression	0.07*	0.09**	0.01	−0.05	0.37***	−0.05	-	-
Parent-Reported								
8. Internalizing Symptoms	−0.02	−0.02	−0.05	−0.05	−0.07*	0.03	−0.14***	-
9. Externalizing Symptoms	0.01	−0.05	0.01	0.02	−0.01	−0.06^†^	0.10**	0.53***

† *p* < 0.10.

* *p* < 0.05.

** *p* < 0.01.

*** *p* < 0.001.

All mother-youth latent PNS-mediated synchrony factors were highly correlated to one another (*r* = 0.94–0.96, *p* < 0.001). Respiration rate synchrony was not related to any of the PNS-mediated functioning (*r* = −0.03–−0.12).

In addition, paired sample t-tests were conducted prior to hypothesis testing to see whether positive and negative affect changed from pre- to post-PCT in accordance with what is expected given that the PCT was designed to induce stress. Affect changed in the expected direction across the PCT. Positive affect decreased, [*t*(170) = 16.83, *p* < 0.001] while negative affect increased [*t*(169) = −2.37, *p* = 0.02] from pre- (*M*_positive_ = 12.44, *SD*_positive_ = 3.69; *M*_negative_ = 5.92, *SD*_negative_ = 2.63) to post-PCT (*M*_positive_ = 10.72, *SD*_positive_ = 3.87; *M*_negative_ = 6.13, *SD*_negative_ = 2.90).

### Aim 1: mother-youth physiological synchrony and youth affect

Higher pre-stressor positive affect predicted lower mother-youth RSA synchrony with a moderate-to-large effect (*B* = −0.01, *p* = 0.01, *β* = −0.42). In the focal analysis, greater mother-youth RSA synchrony during the stressor task predicted higher positive affect after the stressor task with a large effect size, after accounting for pre-stressor positive affect (*B* = 0.01, *p* = 0.003, *β* = 0.50). No other synchrony index was significantly associated with post-stressor positive affect. No synchrony index was significantly associated with post-stressor negative affect after accounting for the corresponding pre-stressor affect indicator (Table 7).

**Table 7:** Unstandardized associations examining relationship between physiological synchrony and youth wellbeing.

Variable	Mean	Respiration Rate Synchrony	RMSSD Synchrony	RSA Synchrony
	Estimate (*SE*)	Estimate (*SE*)	Estimate (*SE*)	Estimate (*SE*)
Child-Reported				
Pre-Stressor Positive Affect	12.44 (0.29)***	0.03 (0.02)	0.05 (0.06)	−0.01 (0.004)**
Post-Stressor Positive Affect	10.74 (0.30)***	−0.01 (0.02)	−0.09 (0.07)	0.01 (0.004)*
Pre-Stressor Negative Affect	6.00 (0.22)***	0.03 (0.03)	0.07 (0.09)	−0.002 (0.01)
Post-Stressor Negative Affect	6.16 (0.23)***	0.01 (0.02)	−0.12 (0.08)^†^	−0.01 (0.01)
Depression Symptoms	7.15 (0.52)***	0.004 (0.01)	−0.01 (0.04)	−0.001 (0.002)
Anxiety Symptoms	50.66 (1.08)***	0.004 (0.004)	0.02 (0.02)^†^	−0.001 (0.001)
Aggression	65.06 (1.97)***	0.001 (0.004)	0.0001 (0.01)	0.0001 (0.001)
Parent-Reported				
Internalizing Symptoms	0.82 (0.07)***	−0.01 (0.08)	−0.15 (0.26)	0.04 (0.01)**
Externalizing Symptoms	5.61 (0.41)***	−0.02 (0.01)^†^	0.003 (0.05)	−0.004 (0.002)*

RMSSD, root mean square of successive differences. Positive and negative affect were covaried in the model, as well as internalizing and externalizing symptoms. Child sex, mother age, race, and PANAS pre-stressor were controlled for in the models.

† *p* < 0.10.

* *p* < 0.05.

** *p* < 0.01.

*** *p* < 0.001.

### Aim 2: mother-youth physiological synchrony and youth-reported youth wellbeing

No physiological synchrony index was significantly associated with youth-reported anxiety, depressive symptoms, or aggression. RMSSD synchrony showed a nonsignificant trend toward greater anxiety symptoms, with a small-to-moderate effect size (*B* = 0.02, *p* = 0.08, *β* = 0.26; Table 7).

### Aim 3: mother-youth physiological synchrony and mother-reported youth wellbeing

Greater RSA synchrony was significantly associated with lower mother-reported externalizing symptoms (*B* = −0.004, *p* = 0.04, *β* = −0.31) and higher mother-reported internalizing symptoms (*B* = 0.04, *p* = 0.003, *β* = 0.48), with moderate-to-large effect sizes. Respiration-rate synchrony showed a marginally significant trend toward lower externalizing symptoms (*B* = −0.02, *p* = 0.06, *β* = −0.32), with a moderate effect size (Table 7).

## Discussion

The current study examined whether mother-youth trend-level physiological synchrony during a laboratory stressor was associated with multiple indicators of youth emotional and behavioral functioning in preadolescence. Overall, the findings did not support a uniform association between greater physiological synchrony and better functioning among youth. Greater RSA synchrony was associated with higher post-stressor positive affect after accounting for pre-stressor positive affect and with lower mother-reported externalizing symptoms. Still, it was also associated with higher mother-reported internalizing symptoms. Associations involving RMSSD and respiration-rate synchrony were otherwise nonsignificant, with two marginal trends. Thus, the findings are most consistent with a context-dependent view in which physiological synchrony cannot be interpreted as inherently adaptive or maladaptive.

### RSA synchrony and youth affect

The clearest finding consistent with an adaptive interpretation of synchrony involved positive affect. Greater mother-youth RSA synchrony during the PCT was associated with higher positive affect in youth after the task, even after accounting for pre-task positive affect. This finding is consistent with prior work linking parent-child synchrony with aspects of more positive emotional functioning (28, 44). However, the present data do not identify the mechanism underlying this association. Although coordinated RSA trajectories may reflect co-regulatory processes, the study did not assess whether mothers regulated youths' physiological responses or whether youth regulated mothers' responses. Accordingly, the finding is best interpreted as an association between greater similarity in mother and youth RSA trajectories and more favorable affective functioning.

The absence of associations between synchrony and post-stressor negative affect also warrants a restrained interpretation. Youth reported relatively low and less variable negative affect both before and after the PCT, which may have limited power to detect individual differences (45). In addition, the PANAS-C negative-affect composite captures several emotions, whereas a cognitive performance stressor may evoke more specific states such as frustration, nervousness, or anger. Prior work using relational stressors and discrete emotions has yielded distinct patterns (28), raising the possibility that associations between physiological synchrony and affect depend in part on the type of stressor and the emotional response assessed. These possibilities should be tested directly rather than inferred from the present null findings.

It is also important not to overinterpret differences across physiological indices. RSA synchrony was associated with post-stressor positive affect, whereas RMSSD and respiration-rate synchrony were not. This pattern could reflect differences in what the measures capture. Still, RSA and RMSSD synchrony were highly correlated in this sample. Therefore, differences in statistical significance across these closely related PNS indices do not, by themselves, demonstrate distinct underlying physiological mechanisms. Replication will be important for determining whether RSA has a uniquely informative association with post-stressor affect.

### Physiological synchrony and internalizing symptoms

The internalizing findings further complicate a uniformly adaptive interpretation of synchrony. Greater RSA synchrony was associated with higher mother-reported youth internalizing symptoms, whereas no synchrony index was significantly associated with youth-reported anxiety or depressive symptoms. RMSSD synchrony showed only a marginal trend with youth-reported anxiety. Because internalizing symptoms and synchrony were assessed concurrently, the direction of association cannot be determined. Greater synchrony could reflect characteristics of dyads in which youth experience more internalizing difficulties, or preexisting youth or family characteristics could shape how mothers and youth respond physiologically during stress. The present design cannot distinguish these possibilities. Future longitudinal and lagged analyses, ideally combined with behavioral observations during the stressor, could test whether observable distress precedes changes in parental physiology or vice versa.

### Physiological synchrony and externalizing symptoms

Greater RSA synchrony was associated with lower mother-reported externalizing symptoms. This finding is consistent with the possibility that some forms of physiological coordination are associated with more adaptive behavioral functioning. Still, it should be interpreted alongside the positive association between RSA synchrony and mother-reported internalizing symptoms. The contrasting directions across two outcomes reported by the same informant suggest that greater synchrony is not simply a marker of better overall adjustment. More broadly, discrepancies across youth- and mother-reported outcomes highlight the value of multi-informant assessment (46–48), particularly because internalizing symptoms may be less observable to parents than externalizing behaviors. Subsequent studies should examine whether these associations replicate when the same constructs are assessed across multiple informants and methods. The findings offer an implication for study design: studies should assess multiple physiological indices, distinguish among forms and timescales of synchrony, and pair physiological data with behavioral measures that can clarify what coordinated physiological change represents within a particular interaction.

### Limitations

Several limitations qualify the results. First, most youth functioning measures were assessed concurrently with the laboratory visit, preventing conclusions about temporal precedence or directionality. Longitudinal research that assesses both synchrony and functioning repeatedly is needed to clarify directionality. The only measures that can be sequenced in time are positive and negative affect, as they were measured before and after the stressor task, providing some indication that synchrony, as expected, is related to affective responses to a stressor, which may have implications in longer-term emotional wellbeing. Second, the study did not include measures of interaction behavior that could distinguish adaptive co-regulation from shared arousal or mutual dysregulation. Third, only mothers participated, limiting generalizability to other caregiver-child relationships (e.g., father-child). The relatively high educational attainment and household income of the sample may also limit generalizability to more socioeconomically diverse families.

The study also focused specifically on trend-level synchrony derived from six one-minute epochs. This approach detects similarity in mothers' and youths' general physiological trajectories across the task. In this study, we were interested in asking whether the similarity in changes in reactivity was associated with wellbeing. However, this analytic approach does not determine whether deviations from the trend were synchronized within mother-youth dyads at the same moment (i.e., concurrent synchrony) or whether moment-to-moment changes in one partner's cardiovascular reactivity influenced subsequent changes in the other's (i.e., lagged synchrony). To investigate these other types of synchrony, more statistical power is required. Also, concurrent and lagged synchrony are typically evaluated at smaller time-scale intervals, therefore trend-level synchrony was the most appropriate choice for evaluating synchrony. If we attempted to evaluate concurrent or lagged synchrony, synchrony may have been obscured. Future research should examine multiple forms of synchrony in brief time-scale intervals within the same sample to determine whether their differential associations with wellbeing are attributable to the interaction context or relationship in which synchrony is measured, or to differences in the methods used to quantify synchrony. In addition, the very high correlations among the PNS synchrony factors make it difficult to infer that differences in their associations with outcomes reflect distinct physiological processes. Future studies should compare multiple synchrony metrics within the same sample and at multiple timescales to determine which findings are robust to measurement approach.

## Conclusion

The current study examined mother-youth trend-level physiological synchrony in response to a stressor and its relation to youth wellbeing during preadolescence. Greater RSA synchrony was associated with higher post-stressor positive affect, lower mother-reported externalizing symptoms, and higher mother-reported internalizing symptoms; most other associations were nonsignificant. This mixed pattern suggests that physiological synchrony should not be treated as a uniformly adaptive marker of co-regulation. Instead, its meaning may depend on the physiological index, the outcome, the developmental period, and the interactional context. Longitudinal, multi-method studies that combine physiology with behavioral observations will be important for clarifying when parent-youth synchrony reflects adaptive regulation and when it reflects other forms of shared responding.

## Data Availability

The datasets presented in this study can be found in online repositories. The names of the repository/repositories and accession number(s) can be found below: https://osf.io/semb4/overview?view_only=ab421428470f497995e824d35f8e5716.

## Footnotes

^1^Rowley CA, Li F, Ruiz M, Cuevas-Kovanis ZO, Ford J, Somers JA, et al. Motherchild physiological synchrony under stress: the role of sensitive parenting. [Unpublished]

## References

[1] Feldman R. Parent-infant synchrony: a biobehavioral model of mutual influences in the formation of affiliative bonds. Monogr Soc Res Child Dev. (2012) 77(2):42–51. 10.1111/j.1540-5834.2011.00660.x

[2] Birk SL Stewart L Olino TM. Parent-child synchrony after early childhood: a systematic review. Clin Child Fam Psychol Rev. (2022) 25(3):529–51. 10.1007/s10567-022-00383-7 35133524

[3] Feldman R. Parent-infant synchrony and the construction of shared timing: physiological precursors, developmental outcomes, and risk conditions. J Child Psychol Psychiatry. (2007) 48(3-4):329–54. 10.1111/j.1469-7610.2006.01701.x 17355401

[4] Xu J Zhang Y Wang H Peng M Zhu Y Wang X. A context-dependent perspective to understand the relation between parent-child physiological synchrony and child socioemotional adjustment. Dev Sci. (2024) 27(6):e13506. 10.1111/desc.13506 38549214

[5] Davis M West K Bilms J Morelen D Suveg C. A systematic review of parent–child synchrony: it is more than skin deep. Dev Psychobiol. (2018) 60(6):674–91. 10.1002/dev.21743 29900545

[6] Gillespie L. It takes two: the role of co-regulation in building self-regulation skills. Young Child. (2015) 70(3):94–6. Available online at: https://www.naeyc.org/resources/pubs/yc/jul2015/rocking-rolling (Accessed June 1, 2026).

[7] DePasquale CE. A systematic review of caregiver-child physiological synchrony across systems: associations with behavior and child functioning. Dev Psychopathol. (2020) 32(5):1754–77. 10.1017/S0954579420001236 33427185

[8] Creavy KL Gatzke-Kopp LM Zhang X Fishbein D Kiser LJ. When you go low, I go high: negative coordination of physiological synchrony among parents and children. Dev Psychobiol. (2020) 62(3):310–23. 10.1002/dev.21905 31410845

[9] Pratt M Apter-Levi Y Vakart A Kanat-Maymon Y Zagoory-Sharon O Feldman R. Mother-child adrenocortical synchrony: moderation by dyadic relational behavior. Horm Behav. (2017) 89:167–75. 10.1016/j.yhbeh.2017.01.003 28131596

[10] Waters SF West TV Mendes WB. Stress contagion: physiological covariation between mothers and infants. Psychol Sci. (2014) 25(4):934–42. 10.1177/0956797613518352 24482403 PMC4073671

[11] Horowitz-Kraus T Gashri C. Multimodal approach for characterizing the quality of parent-child interaction: a single synchronization source may not tell the whole story. Biology (Basel). (2023) 12(2):241. 10.3390/biology12020241 36829518 PMC9952901

[12] Ziegler MG. Psychological stress and the autonomic nervous system. In: Robertson D Biaggioni I Burnstock G Low PAF Paton JFR, editors. Primer on the Autonomic nervous System, 3rd Edn. Amsterdam: Academic Press (2012). p. 291–3. 10.1016/B978-0-12-386525-0.00061-5

[13] Klink ESC Pruessner JC. Investigating the interaction of sympathetic and parasympathetic nervous system under stress and relaxation using guided imagery. Psychological Research of Early Career Scientists. (2023) 1(1):1–30. 10.17605/OSF.IO/HSV6A

[14] Beauchaine TP. Respiratory sinus arrhythmia: a transdiagnostic biomarker of emotion dysregulation and psychopathology. Curr Opin Psychol. (2015) 3:43–7. 10.1016/j.copsyc.2015.01.017 25866835 PMC4389219

[15] Huizinga JD Mathewson KJ Yuan Y Chen JH. Probing heart rate variability to determine parasympathetic dysfunction. Physiol Rep. (2018) 6(10):e13713. 10.14814/phy2.13713 29845773 PMC5974730

[16] Brown RL Chen MA Paoletti J Dicker EE Wu-Chung EL LeRoy AS. Emotion regulation, parasympathetic function, and psychological wellbeing. Front Psychol. (2022) 13:879166. 10.3389/fpsyg.2022.879166 35992409 PMC9381823

[17] Kim HG Cheon EJ Bai DS Lee YH Koo BH. Stress and heart rate variability: a meta-analysis and review of the literature. Psychiatry Investig. (2018) 15(3):235. 10.30773/pi.2017.08.17 29486547 PMC5900369

[18] Moridani MK Mahabadi Z Javadi N. Heart rate variability features for different stress classification. Bratisl Med J. (2020) 121(09):619–27. 10.4149/bll_2020_107 32990009

[19] Shaffer F Ginsberg JP. An overview of heart rate variability metrics and norms. Front Public Health. (2017) 5:258. 10.3389/fpubh.2017.00258 29034226 PMC5624990

[20] Castaldo R Melillo P Bracale U Caserta M Triassi M Pecchia L. Acute mental stress assessment via short term HRV analysis in healthy adults: a systematic review with meta-analysis. Biomed Signal Process Control. (2015) 18:370–7. 10.1016/j.bspc.2015.02.012

[21] Helm JL Miller JG Kahle S Troxel NR Hastings PD. On measuring and modeling physiological synchrony in dyads. Multivariate Behav Res. (2018) 53(4):521–43. 10.1080/00273171.2018.1459292 29683720

[22] Feldman R. Mother-infant skin-to-skin contact and the development of emotion regulation. Adv Psychol Res. (2004) 27:113–31. 10.1097/00001163-200404000-00006

[23] Cera R Mancini M Antonietti A. Relationships between metacognition, self-efficacy, and self-regulation in learning. J Educ Cult Psychol Stud. (2013) 4(7):115–41. 10.7358/ecps-2013-007-cera

[24] Fomina T Burmistrova-Savenkova A Morosanova V. Self-regulation and psychological wellbeing in early adolescence: a two-wave longitudinal study. Behav Sci. (2020) 10(3):67. 10.3390/bs10030067 32164173 PMC7139468

[25] Aldao A Gee DG De Los Reyes A Seager I. Emotion regulation as a transdiagnostic factor in the development of internalizing and externalizing psychopathology: current and future directions. Dev Psychopathol. (2016) 28(4 Pt 1):927–46. 10.1017/S0954579416000638 27739387

[26] Md Din NSB Ahmad M. Emotional regulation on negative affect and aggression: a review. Asian People J. (2021) 4(2):29–44. 10.37231/apj.2021.4.2.281

[27] Tangney JP Baumeister RF Boone AL. High self-control predicts good adjustment, less pathology, better grades, and interpersonal success. J Pers. (2004) 72(2):271–324. 10.1111/j.0022-3506.2004.00263.x 15016066

[28] Woody ML Feurer C Sosoo EE Hastings PD Gibb BE. Synchrony of physiological activity during mother-child interaction: moderation by maternal history of major depressive disorder. J Child Psychol Psychiatry. (2016) 57(7):843–50. 10.1111/jcpp.12562 27090774 PMC5680529

[29] Suveg C Braunstein West K Davis M Caughy M Smith EP Oshri A. Symptoms and synchrony: mother and child internalizing problems moderate respiratory sinus arrhythmia concordance in mother-preadolescent dyads. Dev Psychol. (2019) 55(2):366–76. 10.1037/dev0000648 30507218

[30] Han ZR Gao MM Yan J Hu X Zhou W Li X. Correlates of parent-child physiological synchrony and emotional parenting: differential associations in varying interactive contexts. J Child Fam Stud. (2019) 28(4):1116–23. 10.1007/s10826-019-01337-4

[31] Woltering S Lishak V Elliott B Ferraro L Granic I. Dyadic attunement and physiological synchrony during mother-child interactions: an exploratory study in children with and without externalizing behavior problems. J Psychopathol Behav Assess. (2015) 37(4):624–33. 10.1007/s10862-015-9480-3

[32] Oudekerk BA Allen JP Hessel ET Molloy LE. The cascading development of autonomy and relatedness from adolescence to adulthood. Child Dev. (2015) 86(2):472–85. 10.1111/cdev.12313 25345623 PMC4376599

[33] Koehn AJ Kerns KA. The supervision partnership as a phase of attachment. J Early Adolesc. (2016) 36(7):961–88. 10.1177/0272431615590231

[34] Kerns KA Brumariu LE. Attachment in middle childhood. In: Cassidy J Shaver PR, editors. Handbook of Attachment: Theory, Research, and Clinical applications, 3rd Edn. New York, NY: Guilford Press (2016). p. 349–65.

[35] Hofer MA. Hidden regulators in attachment, separation, and loss. Monogr Soc Res Child Dev. (1994) 59(2-3):192–207. 10.1111/j.1540-5834.1994.tb01285.x 7984161

[36] Borelli JL Margolin G Rasmussen HF. Parental overcontrol as a mechanism explaining the longitudinal association between parent and child anxiety. J Child Fam Stud. (2015) 24(6):1559–74. 10.1007/s10826-014-9960-1

[37] Weschler D. Wechsler Intelligence Scale for Children-Third Edition (WISC-III). San Antonia (TX): The Psychological Corporation (1991).

[38] Watson D Clark LA Tellegen A. Development and validation of brief measures of positive and negative affect: the PANAS scales. J Pers Soc Psychol. (1988) 54(6):1063–70. 10.1037/0022-3514.54.6.1063 3397865

[39] Kovacs M. Children's Depression Inventory Manual. North Tonawanda (NY): Multi-Health Systems (1992).

[40] March JS Parker JDA Sullivan K Stallings P Conners CK. The multidimensional anxiety scale for children (MASC): factor structure, reliability, and validity. J Am Acad Child Adolesc Psychiary. (1997) 36(4):554–65. 10.1097/00004583-199704000-00019 9100431

[41] Buss AH Warren WL. Aggression Questionnaire Manual. Los Angeles (CA): West Psychological Services (2000).

[42] Achenbach TM Rescorla LA. Manual for the ASEBA School-Age Forms and Profiles. Burlington (VT): University of Vermont, Research Center for Children, Youth, and Families (2001).

[43] Muthén LK Muthén BO. Mplus user's guide: Statistical Analysis with Latent variables. 8th ed Los Angeles (CA): Muthén & Muthén (1998-2017).

[44] Kudinova AY Woody ML James KM Burkhouse KL Feurer C Foster CE. Maternal major depression and synchrony of facial affect during mother-child interactions. J Abnorm Psychol. (2019) 128(4):284–94. 10.1037/abn0000411 31045411 PMC7034770

[45] Booy RM Carolan PL. The role of selective attention in the positivity offset: evidence from event-related potentials. PLoS One. (2021) 16(11):e0258640. 10.1371/journal.pone.0258640 34731204 PMC8565729

[46] De Los Reyes A Epkins CC. Introduction to the special issue. A dozen years of demonstrating that informant discrepancies are more than measurement error: toward guidelines for integrating data from multi-informant assessments of youth mental health. Journal of Clinical Child & Adolescent Psychology. (2023) 52(1):1–8. 10.1080/15374416.2022.2158843 36725326

[47] Van Roy B Groholt B Heyerdahl S Clench-Aas J. Understanding discrepancies in parent-child reporting of emotional and behavioural problems: effects of relational and socio-demographic factors. BMC Psychiatry. (2010) 10(1):56. 10.1186/1471-244X-10-56 20637090 PMC2912799

[48] Bajeux E Klemanski DH Husky M Leray E Chan Chee C Shojaei T. Factors associated with parent-child discrepancies in reports of mental health disorders in young children. Child Psychiatry Hum Dev. (2018) 49:1003–10. 10.1007/s10578-018-0815-7 29869765

